# Older Adult Peer Specialists’ Role in Reducing Loneliness Among People With Mental Health Conditions

**DOI:** 10.1093/geroni/igaa057.2288

**Published:** 2020-12-16

**Authors:** Karen Fortuna, George Mois, Jessica Brooks, Amanda Myers, Cynthia Bianco

**Affiliations:** 1 Dartmouth College, Concord, New Hampshire, United States; 2 University of Georgia, Lawrenceville, Georgia, United States; 3 Columbia University, New York, New York, United States; 4 Rivier University, Hudson, Massachusetts, United States

## Abstract

PeerTECH is a peer-delivered and technology-support integrated medical and psychiatric self-management intervention developed by peers. A pre/post trial by our group has shown PeerTECH is associated with statistically significant improvements in self-efficacy for managing chronic disease and psychiatric self-management skills. This presentation will discuss the feasibility and potential effectiveness of using ecological momentary assessments (EMA) with older adults with mental health conditions to allow us to recognize early signs of loneliness and intervene as early as possible in real-world settings. EMA involves repeated sampling of an individual’s behaviors and experiences in real time, real-world environments on the smartphone application. Then, we will discuss the main and interactive effects of loneliness and factors linked to mortality. In conclusion, we will discuss potential effectiveness of PeerTECH with older adults with SMI.

